# Engagement with health research summaries via digital communication to *All of Us* participants

**DOI:** 10.1093/jamia/ocae185

**Published:** 2024-07-25

**Authors:** Janna Ter Meer, Royan Kamyar, Christina Orlovsky, Ting-Yang Hung, Tamara Benrey, Ethan Dinh-Luong, Giorgio Quer, Julia Moore Vogel

**Affiliations:** Scripps Research Translational Institute, La Jolla, CA 92037, United States; Owaves Inc, Encinitas, CA 92024, United States; Scripps Research Translational Institute, La Jolla, CA 92037, United States; Scripps Research Translational Institute, La Jolla, CA 92037, United States; Owaves Inc, Encinitas, CA 92024, United States; Scripps Research Translational Institute, La Jolla, CA 92037, United States; Scripps Research Translational Institute, La Jolla, CA 92037, United States; Scripps Research Translational Institute, La Jolla, CA 92037, United States

**Keywords:** health communication, communication barriers, data science, stakeholder participation

## Abstract

**Objective:**

Summaries of health research can be a complementary way to return value to participants. We assess how research participants engage with summaries via email communication and how this can be improved.

**Materials and Methods:**

We look at correlations between demographic subgroups and engagement in a longitudinal dataset of 305 626 participants (77% are classified as underrepresented in biomedical research) from the *All of Us* Research Program. We compare this against engagement with other program communications and use impact evaluations (N = 421 510) to measure the effect of tailoring communication by (1) eliciting content preferences, (2) Spanish focused content, (3) informational videos, and (4) article content in the email subject line.

**Results:**

Between March 2020 and October 2021, research summaries reached 67% of enrolled participants, outperforming other program communication (60%) and return of results (31%), which have a high uptake rate but have been extended to a subset of eligible participants. While all demographic subgroups engage with research summaries, participants with higher income, educational attainment, White, and older than 45 years open and click content most often. Surfacing article content in the email subject line and Spanish focused content had negative effects on engagement. Video and social media content and eliciting preferences led to a small directional increase in clicks.

**Discussion:**

Further individualization of tailoring efforts may be needed to drive larger engagement effects (eg, delivering multiple articles in line with stated preferences, expanding preference options). Our findings are likely a conservative representation of engagement effects, given the coarseness of our click rate measure.

**Conclusions:**

Health research summaries show promise as a way to return value to research participants, especially if individual-level results cannot be returned. Personalization of communication requires testing to determine whether efforts are having the expected effect.

## Introduction

Health research programs and clinical trials are increasingly focusing on returning value to create more mutually beneficial relationships between study participants and researchers.^1^ This is emblematic of an improvement in the relationship between research participants and researchers—instead of being only passive subjects, returning value is one way to recognize participants as essential stakeholders in and contributors to the research process. Foremost, returning value focuses on study results that apply to the individual’s own health, such as pharmacogenomic results^2^ and treatment and prevention of disease.^3^ However, most studies do not return results to participants^4^ for multiple reasons, including inability to present findings in a way that is meaningful and/or accessible to the participant, lack of resources, lack of regulatory approval, and concerns over results being inconclusive, disappointing, or distressing.^1^^,^^5^

Summaries of relevant health research, including work informed by study data, can be a complementary way to return value to participants. Participants may derive value from health research summaries in several ways. First, summaries that leverage data from the research study may increase the awareness that participants are contributing to impactful research, which has been found to increase charitable donations^6^ and blood donations.^7^ A desire to learn about scientific advances based on study results has been mentioned in focus groups^8^ and in survey work with current and potential research participants.^4^ In addition, summaries may communicate contributions by other participants or highlight work by study staff and leadership, which can engender feelings of shared commitment^9^^,^^10^ and increase perceived trustworthiness and competence.^11^^,^^12^ Third, information in a summary format using simple language can make content a valuable educational resource for participants who want to increase their health literacy^13^ or learn about specific topics that interest them,^4^ such as how lifestyle affects the risk of medical conditions.^2^ Finally, summaries can be tailored for relevance to precision medicine, health equity, and health priorities of communities that have been historically underrepresented in biomedical research (UBR), which can be important for individuals who are motivated to improve representation of such communities in health research.^14^

## Objective

We assess how research participants engage with email communication of health research summaries and how engagement may be improved. We leverage a rich longitudinal dataset of more than 300 000 participants from the *All of Us* Research Program (*All of Us*)^15^ to assess which demographic subgroups are most likely to engage with health research summaries. We then benchmark these engagement metrics against other program communication, including return of results.

While all demographic subgroups engage with health research summaries, open and click rates are lower for certain UBR subgroups, such as participants self-identifying as Black, African American, Hispanic, Latino, or Spanish (17.7% of readership) and those aged 18-45 years (37.3% of readership). We evaluate whether engagement of key UBR subgroups can be improved by tailoring content and delivery in a way that is better aligned with participant preferences. We do this through 4 different initiatives: (1) Content to Spanish participants that is written by native Spanish speakers rather than translated from English and features studies specially curated for Latino and Hispanic communities in the United States. This may increase engagement because participants may consider content about their community relevant for their own decision making or derive positive feelings from seeing their community represented.^16^ (2) Package content in short video format via YouTube and Instagram. This may be appealing to individuals aged 18-25, who show higher self-reported interest in research^17^ and willingness to learn more^18^ in response to videos compared to text. (3) Ask participants to choose what content they want to read about in the following issue and deliver accordingly. This can help participants select content that is closer to their individual preferences.^19^ Participants may also value the choice itself.^20^ (4) Mentioning relevant newsletter topics in the email subject line, which can drive engagement through increased salience.^21^ We assess the impact of these tailoring efforts through a series of well-powered evaluations run between November 2021 and August 2023.

## Materials and methods

### *All of Us* and the *My Medical Minutes* newsletter

*All of Us* is a longitudinal study funded by the National Institutes of Health (NIH) to understand the impact of genetics, lifestyle, and environmental factors on health outcomes, especially those of individuals who have been historically underrepresented in medical research (Table S1). The data generated by the program are available to vetted researchers through a secure data portal that removes personally identifiable information. As of March 2024, the program counts more than 778 000 enrolled participants (87% UBR) and over 9800 researchers across 731 institutions. More than 290 papers have been published using study data,^22^ including the discovery of 275 million novel genetic variants.^23^

Each month participants receive the *My Medical Minutes* (MMM) email newsletter with 3-5 summaries of research covering a range of topics including healthcare access, genomics, health technology, as well as personal stories of people overcoming health challenges or achieving medical breakthroughs. Content is sourced from recently published peer-reviewed journals and health news sites, is summarized at a 7th-grade or lower reading level by a medical copywriter, and is reviewed for scientific accuracy by an MD or PhD. Since June 2021, the newsletter also features summaries of published articles that use *All of Us* study data, guest editor contributions, and personal stories of participants as well as program staff and leadership. Participant feedback can be provided via a dedicated link in each newsletter, is reviewed monthly, and informs the choice of newsletter articles, which are accompanied by a “Reader’s Choice” flag for easy identification (Figure S1). The newsletter is sent in English or Spanish depending on the participant’s declared language preference.

### Retrospective analysis

For each issue, we record whether an email is opened and whether content in the newsletter is clicked (S3 describes data capture and storage in detail). A click is an action by the participant in the newsletter for more information, to share content via social media or to give feedback. In a given issue, the following elements of the newsletter are clickable: 3-4 links within each health research summary for more information, a program announcement directing to a specific study module, links to share an article via social media, a social media post, a link to unsubscribe from future newsletter communications, and a link to leave feedback. Clicking to unsubscribe is not counted towards the click rate in the analysis.

To understand newsletter engagement, we analyzed data of all MMM issues (30 total) sent since newsletter launch in March 2020 to October 2021. In this period, 305 626 unique participants received at least one MMM issue. We have high-level demographic information about participants from the program’s “Basics” survey, such as age bracket and self-identified race and/or ethnicity. Details about the survey, including questions and answer options, can be found at: https://databrowser.researchallofus.org/survey/the-basics. We measure engagement using email opens and the click rate on a given article, assigning a value of 1 if any link in the article is clicked and 0 otherwise. Clicks to share content via social media or to share feedback to the editorial team are excluded for this analysis. Engagement by subgroup is compared using a proportions z-test. Certain demographic subgroups, such as those with educational attainment of grade 12 or below, are combined so that we have a large enough sample size for analysis (Table S2 provides a breakdown of all demographic subgroups).

### Benchmarking

We compare the engagement metrics of the MMM newsletter against 2 other *All of Us* communications: (1) the bi-monthly national newsletter with program updates, staff interviews, and other research opportunities. This goes out to all registered individuals who have not unsubscribed. (2) Genetic return of results, which is a personalized report with information about ancestry, traits, and genetic variants linked to increased disease risk and response to medications. This is sent to participants who have shared a biosample (saliva or blood) with the program and have opted in to receive their results.

### Personalization strategies

The evaluation of the personalization strategies uses a mix of randomized controlled trials (RCTs) and (non-controlled) before/after comparisons that were run between November 2021 and April 2023. We compare groups on newsletter engagement metrics using the rate of email opens and clicks relative to emails delivered. Before/after comparisons were run where an RCT was not possible due to available program resources. To maximize the validity of our results from before/after analyses, we establish a baseline average of opens and clicks across 4-5 MMM issues prior to the new approach being deployed to the relevant target audience. Note that for the tailored Spanish content, we omit the issues of 4/26 and 6/14 from the baseline because these had a clickable program announcement at the top of the newsletter, which was absent from the intervention emails. Instead, we use data from 2 other preceding issues. After establishing the baseline averages, the new strategy is then deployed to everyone in the relevant target group (eg, participants who selected Spanish as their preferred language) for a minimum of 4 issues to reduce the likelihood that the results are driven by specific content of a particular issue. The only exception to this is the evaluation of the preference elicitation effort, which was only deployed in a single issue. The exact number of issues was determined by balancing concerns for interval validity and statistical power with cost considerations for the editorial team to personalize communication. We analyze the average effect on our engagement metrics and compare this against the before baseline to determine the impact of the personalized material. The sample sizes for these efforts include the full available cohort that meet the criteria for the respective tailoring effort (eg, participants aged 18-45). For the preference elicitation effort, the sample size is restricted to a subset of participants who engage with the program through a technology platform (CareEvolution) that has the capability to implement this type of personalized communication. All statistical tests are run using a proportions z-test without demographic covariates and results are reported as significant if they have a *P*-value of .05 or lower.

The different personalization strategies are as follows:

1. Preference elicitation and feedback: Readers were prompted to choose what content they wanted to receive in the next issue (genetics, sleep, or heart health). Participants who made a selection received a tailored newsletter the following month with an article on their chosen topic along with a note reminding them of their choice (Figure S2). If participants did not make a selection, they would receive a standard issue. This was tested with 30 635 participants between July and August 2023.
2. Content for Spanish speakers: Instead of receiving a Spanish translation of the English version of the newsletter, new content was created by a dedicated Spanish-speaking editorial team and selected with relevance for Latino and Hispanic communities in mind. In addition, the newsletter featured Spanish guest editors and researcher profiles. A total of 11 590 participants who had set Spanish as their preferred language for program communication received 4 issues of the new Spanish newsletter between July and September 2022.
3. Content for participants aged 18-45: Instead of static images and text-based articles, these participants received summaries in the form of a YouTube video and an Instagram post. A total of 107 968 participants received this updated newsletter across 5 issues between August 2022 and April 2023.
4. Displaying topics of interest in the email subject line: Participants were randomly allocated to either receive the generic email subject line of the format “Week of [month] [day]” (eg, “Week of November 2”) or a subject line of the format “[Topic 1], [Topic 2] and more” (eg, “COVID, cognitive health and more”). The specific topics reflect newsletter content of that issue and, where possible, were selected based on article types that scored high on engagement in the retrospective analysis (Table S4). This RCT was run with 271 317 participants between November 2021 and February 2022. Participants stay in their groups for 3 consecutive sends.

The study was reviewed and deemed exempt from Institutional Review Board approval by the *All of Us* Research Compliance Branch (RCB-2023-NHSR001) on the grounds of not being human subjects research. It received programmatic approval and oversight from *All of Us*.

## Results

### Newsletter readership and impact

Between the newsletter launch in March 2019 and March 2024, *All of Us* sent 74 MMM issues. This included more than 20 million unique sends, reaching over 533 500 participants who reflect the diversity of the program (Table 1). A total of 930 participants have shared feedback and suggestions for articles, of which 79% are UBR.

**Table 1.** Breakdown of the newsletter readership by UBR categories for those that are enrolled in *All of Us* and have completed the demographic survey (“Basics”).

UBR category	Count readership	Proportion readership	Representation in program
Self-identified race/ethnicity	185 705	39.2%	41.4%
Age	146 707	31.0%	27.5%
Educational attainment	25 195	5.3%	6.8%
Income	97 237	20.6%	33.7%
Sex	3783	0.8%	0.1%
Gender identity	6816	1.4%	1.5%
Sexual orientation	66 231	14.0%	9.5%
UBR overall	408 886	76.6%	84.7%

Note: Participants can belong to multiple UBR categories.

Articles fall into 11 high-level topic categories (Table 2). In addition, the newsletter has featured 24 articles using *All of Us* data, which generated 117 985 clicks (average of 4916 clicks per article) for more information. The 6 spotlights on *All of Us* participants, staff, and leadership generated 9259 clicks to the research program’s website for more information.

**Table 2.** Types of content featured across 74 English issues of MMM newsletter between March 25, 2019, to March 14, 2024.

Topic category	Subcategory	Article count
Aging	Healthy Aging, Alzheimer's Disease, Cognitive Health, Dementia	12
Medical Conditions	Cancer, Diabetes, Heart Disease, Heart Health, Irritable Bowel Syndrome, Infectious Disease, Stroke, and Other Conditions	39
COVID	COVID-specific treatments and trends; includes Long COVID	41
Diversity	Racial, Ethnic, Cultural, Sexual, and Gender Diversity	26
Innovation	Artificial Intelligence, FDA Approvals, Medical Devices, Pharmaceuticals, and Other Technology	32
Lifestyle	Fitness, Mental Well-being, Nutrition, Relationships, Sleep, Stress, Weight Management	106
Children’s Health (added 02/2024)	Pediatrics, Neonatology, Adolescent Health, Education and Schools	2
Personal Story	Human interest stories about people overcoming health issues or making remarkable advances in healthcare	68
Precision Medicine	Genomics, Gene Therapies, Pharmacogenomics	38
Public Health	Environment, Environmental Health, Global Health, Municipal Health, Social Determinants of Health, Health Access, Community Health	27
Women’s Health	Obstetrics and Gynecology	19
*All of Us* research (added 06/2021)	Research articles using *All of Us* data via the Researcher Workbench	24
*All of Us* spotlights (added 06/2021)	Features showcasing the work of people within the *All of Us* consortium	6

Note: A single article can have multiple labels.

### Benchmarking against other program communication and return of results

Engagement metrics between MMM and the national program newsletter are roughly comparable (Table 3). Both have an average open rate of 29.5% in the period between March 2020 and October 2021 and a content click rate of 2% for the MMM newsletter and 3.5% for the national newsletter. Looking across all issues in that period, the MMM newsletter reached a higher proportion of unique participants than the national newsletter (73.5% compared to 66.8% of those contacted), though engaged fewer unique participants as measured by clicks (16.9% for the MMM newsletter compared to 18.5% for the national newsletter). Approximately 9% of enrolled participants who received both communications engaged only with MMM newsletter and not the national newsletter.

**Table 3.** Engagement metrics of the *My Medical Minutes* newsletter compared to the national program newsletter and return of genetic results.

Communication	*My Medical Minutes* **newsletter**	National program newsletter	Return of genetic results
Time period	Mar 2020–Oct 2021	Mar 2020–Oct 2021	Nov 2020–May 2024
Number of issues	30	11	1
Contacted participants (% of eligible)	305 626 (91.2%)	375 847 (89.3%)	275 097 (49.5%)
Average view rate^a^	29.5%	29.5%	63.0%
Average click rate	2.0%	3.5%	NA
Unique participants viewing (% of contacted)	224 635 (73.5%)	251 066 (66.8%)	173 359 (63.0%)
Unique participants viewing (% of eligible)	224 635 (67.1%)	251 066 (59.6%)	173 359 (31.2%)
Unique participants clicking (% of contacted)	51 651 (16.9%)	69 532 (18.5%)	NA

Note: We cap the timeframe for the newsletter engagement metrics to October 2021, after which the email open rate became less reliable (see “Discussion” section for details).

a “Views” are considered email opens for *My Medical Minutes* and accessing the report in the portal for genetic results.

For genetic results, the uptake is more than twice as high compared to any single issue of the newsletter: 63.0% of participants who are sent a personalized report access it. However, the number of contacted participants is substantially lower: 49.5% of participants who submitted a biosample were issued a report since the first release of return of results in November 2020 and May 2024. Taken together, the reach of the communication relative to participants who are eligible is 31.2% for return of genetic results, compared to 67.1% for MMM newsletter and 59.6% for the national program newsletter.

### Engagement metrics by demographic subgroup

Open and click rates differ substantially by demographic subgroup across the 30 newsletter issues (Table S3). All differences reported are statistically significant at a *P*-value of .001 or less, unless otherwise noted.

**Figure 1.**
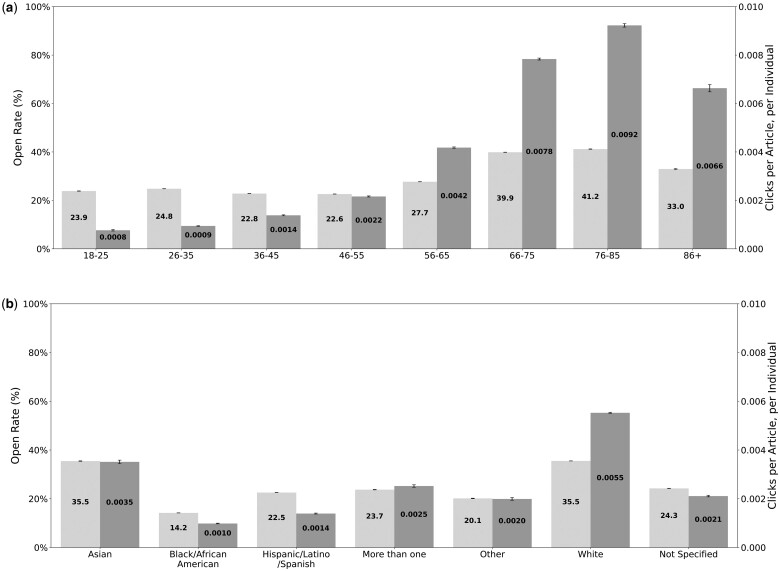
Open (light gray) and click rates (dark gray) across age and race/ethnicity subgroups in the top and bottom panel, respectively. Confidence intervals are shown in black at the top of each bar.

Open rates increase linearly with income and educational attainment. Participants with annual income of less than $25 000 open the newsletter 16.1% of the time, on average, compared to 40.2% for those earning $100 000 or more. For educational attainment, those who completed up to 12th grade open the newsletter 9.1% of the time, on average. The open rate then increases steadily to 16.6% for those with a high school diploma, 25.3% for those who completed some college, up to 35.8% for college graduates, and 41% for those with an advanced degree. For age, participants between the ages of 66 and 85 are most likely to open the newsletter (40.2%). Participants aged 86 and up open the newsletter 33% of the time, followed by participants aged 56-65 (27.7% open rate) and participants between 18-55 years (23.5% open rate) (Figure 1). Participants self-identifying as Asian or White are most likely to open the newsletter (35.5%, on average, *P* = .54), compared to Hispanic, Latino, or Spanish (22.5%) and Black or African American (14.2%). Those self-identifying with more than one race open the newsletter 23.7%, on average (Figure 1).

Click rates vary with the same directionality as open rates, with differences in clicks being largest when participants are grouped by educational attainment, age, income, and racial and/or ethnic background. Specifically, those with an advanced degree are 15 times more likely to click on content compared to those who completed up to grade 12 and 7 times more likely to click compared to those who completed high school. Participants aged 66 or older are 10 times more likely to click on content compared to those aged 18-35 years (Figure 1). Participants with an annual income of $75 000 or higher are 7 times more likely to click than those with an income of less than $10 000 and 3 times as likely to click as participants with an income between $10 000 and $25 000. White participants are 5 times more likely to click than Black, African Americans, Hispanic, Latino, or Spanish, and 1.5 times more likely to click compared to Asian participants (Figure 1). Women are 1.5 times more likely to click on content compared to men and twice as likely to click compared to participants classified as non-binary. The only exception where open rates and click rates diverge is for gender identity, where we see women clicking more often than men and non-binary participants, but no difference in the likelihood of opening the email.

### Impact of tailored content initiatives on engagement

1. When participants were invited to vote for their preferred content, 1631 (5.3%) selected a topic for their next issue. Of those that did, 28.3% clicked on content in the following issue, compared to 24.6% across the 5 issues in the baseline comparison (*P* = .71). Across all participants, the click rate for the issue following the elicitation of content preferences was 4.5% compared to 4.9% in prior issues (*P*=.84). This suggests that the elicitation did not increase engagement overall. However, of those that made a content choice, 38% were not previously engaged in any of the baseline issues. This suggests that the elicitation is somewhat effective at engaging participants that otherwise would not interact with newsletter content.
2. Participants receiving Spanish focused content clicked on newsletter articles at an average rate of 0.55%. This click rate is lower than in the baseline comparison (0.84%) (*P*=.08).
3. Participants aged 18-45 receiving short video content rather than text-based articles were directionally more likely to click content compared to the baseline (0.66% compared to 0.49%, *P*=.08). This represents an increase of approximately 200 participants engaging with the newsletter content that were not previously. We see the directional increase in clicks on the video content compared to the standard written format across each age bracket under 45 (18-25, 26-35, and 36-45).
4. Receiving information about newsletter topics in the subject line (eg, “Cognitive health”) reduces the email open rate compared to the standard subject line. Across the 3 sends, the reduction in the open rate is 1.3 percentage points (3.5% relative effect size, *P*<.01) with a lower and upper bound of −0.8 to −2.1 percentage points across the sends, respectively. The average impact on the click rate is −0.02 percentage points. Even though the average effect of communicating the topic in the subject line is negative for participants overall, it could be that the impact is positive for participants who find those topics engaging. We draw on the insights from the retrospective analysis to look at the treatment effects of the demographic subgroups that were most likely to engage with content about aging (cognitive health), medical conditions (lung cancer) and precision medicine. Even for the demographic subgroups that showed stronger engagement with these topics *a priori*, communicating content about those topics in the subject line had a clear negative effect on open rates. This holds for all demographic subgroups and topics tested (Table S6).

## Discussion

Over the last 5 years, the MMM newsletter has returned health research summaries, including *All of Us* articles and spotlights of participants, staff, and leadership, to more than 533 000 participants. The reach of the communication outperforms that of the program’s national newsletter (67.1% compared to 59.6%) and return of individual genetic results (31.2%). While the majority of participants (63.0%) view their individual results once these are released to them, the reports have been made available to only a subset of eligible participants (49.5%). This highlights an important tradeoff in returning value to participants via program communication: while return of results have high appeal, it is challenging to release the information to large participant cohorts in a short timeframe. In addition, in *All of Us*, participants can only access their personalized reports after completing a consent module and several informing loops, which may deter some participants. By contrast, health research summaries can be sent more frequently and are easier to scale. They can also be appealing for smaller research studies that may not be able to communicate relevant program updates at a regular cadence.

While we see broad engagement with the summaries across the participant population, there are substantial differences between demographic subgroups, including UBR, with participants with higher income, educational attainment, White, and older than 45 years opening and clicking content most often. Efforts to increase engagement by tailoring content to specific subgroups saw mixed results. Two initiatives, surfacing article content in the email subject line and Spanish focused content, had a negative effect. Video and social media content and asking participants what topic they want to see featured in the following issue directionally increased clicks, but this was not statistically significant. In addition, only a relatively small subset (5.3%) of participants responded to the elicitation prompt. This indicates that the personalization efforts we undertook were not sufficient to have a measurable positive impact on engagement. Future tailoring efforts should be more comprehensive to drive larger engagement effects, such as delivering multiple articles/issues in line with stated preferences, broadening the options of personalized content to better accommodate different content interests, and/or a more sustained social media presence. Discussions with *All of Us* participants at Healthcare Provider Organizations (HPOs) reveal that many have a specific reason for joining the study (eg, a disease that runs in their family or a desire to represent an underrepresented community) and delivering program information linked to that motivation may improve engagement. Studies that are more narrow in focus may already select for participants with similar interests and could find this easier to achieve. Future work can also look at whether summaries of study data outperform general health summaries in driving engagement, since participants may feel that they have directly contributed to the result.^6^

To our knowledge, our work is the first to analyze engagement with health research summaries in a large and longitudinal dataset of more than 300 000 participants, of whom 77% are UBR (34.8% is UBR on race/ethnicity). The size of the dataset in combination with the variety of content in the newsletter allows us to benchmark engagement against program communication, analyze engagement for specific demographic subgroups, and evaluate the impact of new delivery mechanisms and content types, such as social media videos. Second, our work contributes robust empirical evidence to a scarce literature on the effects of content tailoring^24–26^ and engagement with research program communication other than return of results. Since we tested different strategies using well-powered evaluations, our findings, including those with null and negative effects, can guide practitioners and future research towards more effective personalization approaches. For example, our results suggest there is little difference between high-quality translations and in-language de novo writing, at least for content written in English and translated to Spanish. Finally, our work highlights the importance of testing engagement strategies before deployment at scale and provides a template for how future efforts can be evaluated.

Our findings are subject to several limitations. Most importantly, the email open rate became less reliable as an engagement measure in September 2021 when Apple implemented a system change that would always record an email as opened even if it was not.^27^ This change affected the last few issues of our retrospective analysis, which ran up until October 2021, and our subject-line RCT, which ran between November 2021 and February 2022. As not to exclude certain participants based on device usage, we opt to use the click rate as our primary measure for the evaluation of personalization efforts starting in 2022. However, the coarseness of the click rate as a primary measure for engagement is a major drawback. Since the health research findings are intentionally presented as short summaries written at a low reading grade level, it is likely that many participants consume content without following up with a click to read more. As such, a failure to observe a click does not mean that the participant was not engaged. The click rate likely represents a very conservative estimate of actual engagement, which makes it more difficult to identify improvements to the participant experience and may understate the importance of improvements in absolute terms (eg, an increase by 100 clicks may represent a much larger increase in article consumption by the broader readership). As such, future work could repeat the preference elicitation and social media content initiatives, which had directionally positive results, with a larger sample size or in settings with more granular usage data, such as time spent on a study app. A second limitation is that our retrospective analysis looks at engagement separately for each demographic subgroup, even though certain characteristics, such as educational attainment and income, are very likely correlated. Future work can look to expand this analysis, especially if more granular engagement metrics are available.

## Conclusion

Our results can guide other research programs on how health research summaries, including those using study data, can be returned to participants in the format of an email newsletter and which demographic subgroups may be hardest to engage. Our results reinforce the importance of testing tailoring efforts, to ensure that they are having the desired effect and an effect size in proportion to the resources required to create tailored experiences. Future work should build even more individually tailored approaches, including consideration of intersectionality, with the goal of improving inclusion of participants currently underrepresented in clinical research.

## Acknowledgments

We gratefully acknowledge *All of Us* Research Program participants. We also acknowledge the collaboration with Isa Rector and Emily L. Chen and the support of colleagues at the National Institutes of Health, Vibrent Health, Wondros, Owaves, CareEvolution and Scripps Research who helped make this work possible.

## Supplementary Material

ocae185_Supplementary_Data

## Data Availability

We can share aggregate data and codebooks used for the analysis upon request. Codebooks and aggregate data underlying this article will be shared on reasonable request to the corresponding author. We may be able to release additional data after consultation with the research program’s IRB.

## References

[1] Downey AS , Busta ER, Mancher M, Botkin JR (eds.). Returning Individual Research Results to Participants: Guidance for a New Research Paradigm. The National Academies Press; 2018. 30001048

[2] Wilkins CH , Mapes BM, Jerome RN, Villalta-Gil V, Pulley JM, Harris PA. Understanding what information is valued by research participants, and why. Health Aff (Millwood). 2019;38(3):399-407. 30830824 10.1377/hlthaff.2018.05046 PMC6706772

[3] Shalowitz DI , Miller FG. Communicating the results of clinical research to participants: attitudes, practices, and future directions. PLoS Med. 2008;5(5):e91. 18479180 10.1371/journal.pmed.0050091 PMC2375946

[4] Long CR , Stewart MK, Cunningham TV, Warmack TS, McElfish PA. Health research participants’ preferences for receiving research results. Clin Trials. 2016;13(6):582-591. 27562368 10.1177/1740774516665598 PMC5286914

[5] Schroter S , Price A, Malički M, Richards T, Clarke M. Frequency and format of clinical trial results dissemination to patients: a survey of authors of trials indexed in PubMed. BMJ Open. 2019;9(10):e032701. 10.1136/bmjopen-2019-032701 PMC6803145 31636111

[6] Cryder CE , Loewenstein G, Scheines R. The donor is in the details. Org Behav Hum Decis Process. 2013;120(1):15-23.

[7] Shehu E , Veseli B, Clement M, Winterich KP. Improving blood donor retention and donor relationships with past donation use appeals. J Serv Res. 2023;27(3):346-363.

[8] Cook S , Mayers S, Goggins K, et al Assessing research participant preferences for receiving study results. J Clin Transl Sci. 2020;4(3):243-249. 10.1017/cts.2019.427 PMC7348009 32695496

[9] Croson RT. Theories of commitment, altruism and reciprocity: evidence from linear public goods games. Econ Inquiry. 2007;45(2):199-216.

[10] Fischbacher U , Gächter S. Social preferences, beliefs, and the dynamics of free riding in public goods experiments. Am Econ Rev. 2010;100(1):541-556.

[11] Buell RW , Kim T, Tsay CJ. Creating reciprocal value through operational transparency. Manag Sci. 2017;63(6):1673-1695.

[12] Buell RW , Norton MI. The labor illusion: how operational transparency increases perceived value. Manag Sci. 2011;57(9):1564-1579.

[13] Bader M , Zheng L, Rao D, et al Towards a more patient-centered clinical trial process: a systematic review of interventions incorporating health literacy best practices. Contemp Clin Trials. 2022;116:106733. 35301134 10.1016/j.cct.2022.106733 PMC9196949

[14] Danila MI , Allison JJ, Goins KV, et al Development of a multi-component intervention to promote participation of Black and Latinx individuals in biomedical research. J Clin Transl Sci. 2021;5(1):e134. 34367678 10.1017/cts.2021.797 PMC8327553

[15] All of Us Research Program Investigators. The “*All of Us*” Research Program. New Engl J Med. 2019;381(7):668-676. 31412182 10.1056/NEJMsr1809937 PMC8291101

[16] Sharot T , Sunstein CR. How people decide what they want to know. Nat Hum Behav. 2020;4(1):14-19. 31932690 10.1038/s41562-019-0793-1

[17] Cowdery JE , Powell JH, Fleming YA, Brown DL. Effectiveness of a short video-based educational intervention on factors related to clinical trial participation in adolescents and young adults: a pre-test/post-test design. Trials. 2019;20(1):7. 30606224 10.1186/s13063-018-3097-2 PMC6318898

[18] En TC , Yee YZ, Zhi WCS, Shaharuddin S, Appalasamy JR. Contextual and culturally appropriate video narratives: a potential health promotion tool for young adults. Health Promot J Austr. 2023;34(4):791-798. 36529522 10.1002/hpja.687

[19] Kelly CA , Sharot T. Individual differences in information-seeking. Nat Commun. 2021;12(1):7062. 34862360 10.1038/s41467-021-27046-5 PMC8642448

[20] Deci EL , Ryan RM. The" what" and" why" of goal pursuits: human needs and the self-determination of behavior. Psychol Inq. 2000;11(4):227-268.

[21] Bordalo P , Gennaioli N, Shleifer A. Salience. Annu Rev Econ. 2022;14(1):521-544.

[22] All of Us Research Hub. Accessed June 2024. https://www.researchallofus.org/publications/

[23] The All of Us Research Program Genomics Investigators. Genomic data in the *All of Us* Research Program. Nature. 2024;627(8003):340-346. 38374255 10.1038/s41586-023-06957-x PMC10937371

[24] Lambrecht A , Tucker C. When does retargeting work? Information specificity in online advertising. J Market Res. 2013;50(5):561-576.

[25] Kuiken J , Schuth A, Spitters M, Marx M. Effective headlines of newspaper articles in a digital environment. Digit Journal. 2017;5(10):1300-1314.

[26] Banerjee A , Urminsky O. The language that drives engagement: a systematic large-scale analysis of headline experiments; 2023. https://papers.ssrn.com/sol3/papers.cfm?abstract_id=3770366

[27] Apple Newsroom. “Apple advances its privacy leadership with iOS 15, iPadOS 15, macOS Monterey, and watchOS 8”. Accessed March 2024. https://www.apple.com/uk/newsroom/2021/06/apple-advances-its-privacy-leadership-with-ios-15-ipados-15-macos-monterey-and-watchos-8/

