# Supplementary material for: Engagement with health research summaries via digital communication to All of Us participants
Source: J Am Med Inform Assoc. 2024 Jul 25;31(12):2908–15. doi: 10.1093/jamia/ocae185 (PMC11631099; doi:10.1093/jamia/ocae185)
Supplement: ocae185_Supplementary_Data [file ocae185_supplementary_data.pdf]

Supplementary Material: Engagement with health research summaries via digital communication to *All of Us* participants

Author list: Janna Ter Meer[1 *], Royan Kamyar[2], Christina Orlovsky[1], Ting-Yang Hung[1], Tamara Benrey[2], Ethan Dinh-Luong[1], Giorgio Quer[1 #], Julia Moore Vogel[1 #]

Institutions:
1. Scripps Research Translational Institute, La Jolla 92037, California, USA
2. Owaves, Inc., 111 C Street, Encinitas, CA, 92024

* Corresponding author
# Co-senior authors

1. UBR definitions

UBR category	Program definition
Self-identified race and ethnicity	American Indian or Alaska Native (AIAN); Asian; Black, African or African American; Hispanic, Latino or Spanish (H/L/S); Middle Eastern or North African (MENA); Native Hawaiian or Other Pacific Islander (NHPI); or More than one race (2+) / Multi-Ancestry
Age	12-17, 65-74, or 75+ years
Income	<$25,000 a year
Education	Less than a High School graduate
Sex	Intersex
Gender identity	Non-Binary; Transman/Transgender Man/FTM; Transwoman/Transgender Woman/MTF; Genderqueer; Genderfluid; Gender variant; Two-Spirit; Questioning or unsure of your gender identity
Sexual orientation	Gay; Lesbian; Bisexual; Queer; Polysexual, omnisexual, sapiosexual or pansexual; Asexual; Have not figured out or are in the process of figuring out your sexuality; Mostly straight, but sometimes attracted to people of your own sex; Do not use labels to identify yourself; Do not know the answer
Disability	With disabilities - Physical disabilities and/or Cognitive disabilities
Rural	Any ZIP code where more than 50% of its population resides in either a Non-Metro County and/or a rural Census Tract was included in the list.
Health care access *	Participant responds to ONE of the following questions with the answer in brackets:<br>● Are you covered by health insurance or some other kind of health care plan? (No)<br>● Is there a place that you USUALLY go to when you are sick or need advice about your health? (Emergency Room)<br>● Have you delayed getting care for any of the following reasons in the PAST 12 MONTHS? You couldn't afford the copay (Yes); Your deductible was too high/or could not afford the deductible (Yes); You had to pay out of pocket for some or all of the procedure (Yes); You live in a rural area where the distance to the healthcare provider is too far (Yes)<br>● During the past 12 months, was there any time when you needed any of the following, but didn't get it because you couldn't afford it? Prescription medicines (Yes); Mental health care or counseling (Yes) Emergency care (Yes); Dental care (Yes); Eyeglasses (Yes); Health provider (Yes); To see a specialist (Yes); Follow-up care (Yes)<br>● During the past 12 months, were any of the following true for you? You skipped medication doses to save money (Yes); You took less medicine to save money (Yes); You delayed filling a prescription to save money (Yes)<br>Alternatively, the participant responds to TWO of the following (sub)questions with an answer provided in brackets:<br>● Have you delayed getting care for any of the following reasons in the PAST 12 MONTHS? Didn't have transportation (Yes);Couldn't get time off work (Yes); Couldn't get child care (Yes); Couldn't get elderly care (Yes)<br>● How often have you either delayed or not gone to see doctors or health care providers because they were different from you in any of these ways? (Always, Most or Some of the time)
Overall	If the participant matches one of the demographic characteristics for UBR inclusion as listed above.

Participants are classified as UBR based on their responses to the Basics survey, available at: https://www.researchallofus.org/data-tools/survey-explorer/the-basics-survey/;
In addition to the Basics survey, UBR by health care access is also established using responses from the Healthcare Access and Utilization Survey. Available at: https://www.researchallofus.org/data-tools/survey-explorer/healthcare-access-utilization-survey/

Table S1: UBR definitions

2. Layout of the MMM newsletter

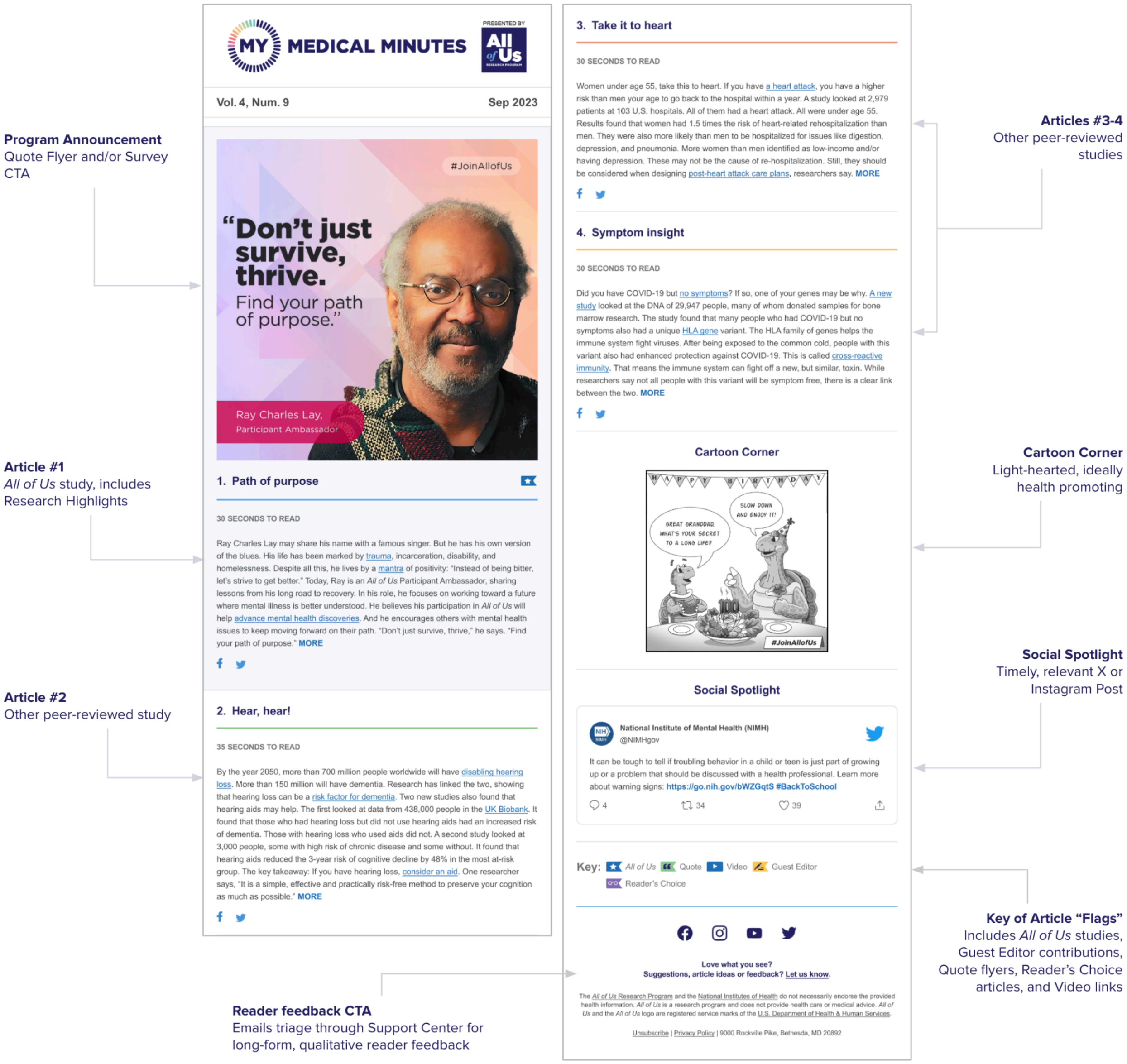

Figure S1: Layout of the MMM newsletter.

MY MEDICAL MINUTES

PRESENTED BY All of Us RESEARCH PROGRAM

Vol. 4, Num. 8 Aug 2023

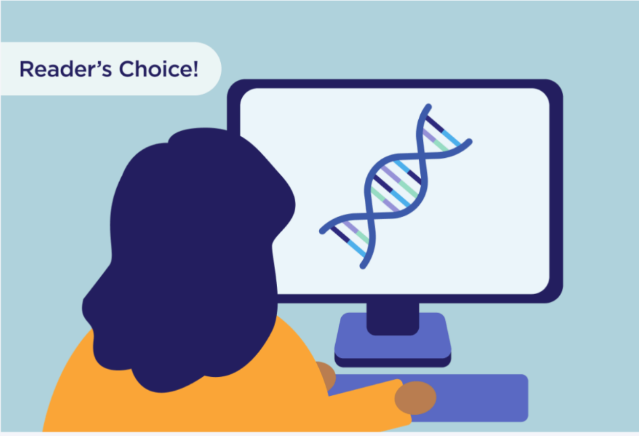

In last month's My Medical Minutes, we asked what you would like to read about: genetics, heart health, or sleep. You chose **genetics**! This month, you'll see a genetics article in the top spot.

We hope you enjoy learning more about your chosen topic and continue to share your thoughts on how we can keep providing content of interest to you.

All of Us

1. Known genomes

30 SECONDS TO READ

Less than 1%. That's the amount of DNA that makes you different from other people. What may seem small can make a huge impact. That's why genomics experts have studied the human genome for decades, looking at small changes that affect health and disease. Now, they have achieved a new milestone. They created a more diverse and comprehensive reference. They call it the pangenome. It adds more than 100 million new bases of DNA. It also includes sequencing from 47 people. Most of our previous knowledge came from DNA information of only one person. By using data from more people, the new reference becomes more representative of more people. This makes it more useful for research on disease risk, prevention, and treatment. The goal of the project is to sequence the genome of 350 people by mid-2024. **MORE**

Figure S2: The personalized newsletter through a "Reader's Choice" segment for participants that selected a topic in the previous issue.

3. Data capture and storage

Data on email opens, clicks and unsubscribes is captured by All of Us via Iterable (Participant Technology Systems Center via Vibrent Health) and MyDataHelps (The Participant Center via CareEvolution) and released to Scripps Research as a consortium partner under its award U24 OD023176 and 1OT2OD035580-01. For the retrospective analysis, All of Us and its IRB gave an exemption to analyze MMM email open, click and unsubscribe data alongside specific high-level demographic categories as captured by the Basics survey in the research program (available at: https://www.researchallofus.org/data-tools/survey-explorer/the-basics-survey/). De-identified datasets were stored on Scripps Research servers and analyzed using Python.

4. Demographic breakdown of MMM readership

Demographic category	Count	Percent
**Self-identified Race and ethnicity**		
Black	61,408	11.5%
Hispanic	58,802	11.0%
Asian	16,999	3.2%
American Indian / American Native	4,433	0.8%
Middle Eastern / North African	2,581	0.5%
Native Hawaiian / Other Pacific Islander	418	0.1%
More than one	36,212	6.8%
White	279,975	52.5%
None of these	4,852	0.9%
Prefer not to answer	2,407	0.5%
Not available *	65,441	12.3%
**Age**		
18-25	25,301	4.74%
26-35	79,692	14.94%
36-45	92,088	17.26%
46-55	86,550	16.22%
56-65	101,969	19.11%
65-75	97,111	18.20%
76-85	43,135	8.08%
86 and over	6,461	1.21%
Not available *	1,259	0.24%
**Educational attainment**		
Never Attended	292	0.1%
One Through Four	1,573	0.3%
Five Through Eight	5,325	1.0%
Nine Through Eleven	18,005	3.4%
Twelve Or GED	71,216	13.3%
College One to Three	125,622	23.5%
College Graduate	121,505	22.8%
Advanced Degree	120,762	22.6%
Prefer not to answer	2,276	0.4%
Not available *	66,987	12.6%
**Income**		
Less 10k	47,783	9.0%
10k-25k	49,454	9.3%
25k-35k	34,552	6.5%
35k-50k	41,725	7.8%
50k-75k	58,564	11.0%
75k-100k	47,373	8.9%

100k-150k	59,312	11.1%
150k-200k	28,705	5.4%
More than 200k	38,090	7.1%
Prefer not to answer	46,478	8.7%
Not available *	81,520	15.3%
**Sex ****		
Female	38445	56.1%
Male	15303	22.3%
Intersex	28	0.0%
None Of These	16	0.0%
Prefer not to answer	59	0.1%
Not available *	14674	21.4%
**Gender identity**		
Woman	296,806	55.6%
Man	166,062	31.1%
Non-Binary	2,470	0.5%
Transgender	923	0.2%
Multiple	2,712	0.5%
Additional options	711	0.1%
Prefer not to answer	650	0.1%
Not available *	63,232	11.9%
**Sexual orientation ****		
Gay	1,324	1.9%
Bisexual	4,190	6.1%
Lesbian	1,003	1.5%
Straight	44,916	65.5%
Multiple	425	0.6%
Prefer not to answer	516	0.8%
Not available *	16,151	23.6%

* Participants receive a value of ‘Not available’ if they chose to skip the question or if they have not completed the Basics survey.
** Data only available from the participants on the CareEvolution platform (12.8% of total readership).

Table S2: Detailed demographic breakdown of the readership of *My Medical Minutes* as of March 2024.

5. Retrospective analysis on open and click rates on MMM articles

	N (% of total in category)	Open rate % (se)	Clicks per article (se)
**Overall cohort**	**305,626**		
**Age**			
18-25	17,912 (5.9%)	23.857 (0.061)	0.0008 (0.0000)
26-35	52,446 (17.2%)	24.806 (0.039)	0.0009 (0.0000)
36-45	43,341 (14.2%)	22.806 (0.039)	0.0014 (0.0000)
46-55	52,940 (17.3%)	22.624 (0.037)	0.0022 (0.0000)
56-65	61,386 (20.1%)	27.690 (0.036)	0.0042 (0.0000)
66-75	51,406 (16.8%)	39.873 (0.042)	0.0078 (0.0000)
76-85	21,898 (7.2%)	41.181 (0.069)	0.0092 (0.0001)
86+	3,611 (1.2%)	32.954 (0.166)	0.0066 (0.0002)
Not specified	684 (0.2%)	43.209 (0.532)	0.0050 (0.0004)
**Gender identity**			
Man	105,480 (34.5%)	27.454 (0.027)	0.0027 (0.0000)
Woman	168,476 (55.1%)	29.789 (0.022)	0.0046 (0.0000)
Non-binary	2,469 (0.80%)	26.922 (0.186)	0.0021 (0.0001)
Not specified	29,201 (9.6%)	24.291 (0.055)	0.0020 (0.0000)
**Self-identified Race/Ethniciy**			
Asian	9,995 (3.3%)	35.464 (0.097)	0.0035 (0.0001)
Black/African American	48,043 (15.7%)	14.197 (0.031)	0.0010 (0.0000)
Hispanic/Latino/Spanish	36,654 (12.0%)	22.548 (0.043)	0.0014 (0.0000)
Native Hawaiian/Pacific Islander	318 (0.1%)	17.949 (0.458)	0.0006 (0.0001)
More than one race	10,639 (3.5%)	23.727 (0.081)	0.0025 (0.0001)
Other race	7,688 (2.5%)	20.228 (0.091)	0.0020 (0.0001)
White	162,746 (53.3%)	35.525 (0.024)	0.0055 (0.0000)
Not specified	29,543 (9.7%)	24.254 (0.053)	0.0021 (0.0000)
**Education level**			
0-12	15,933 (5.2%)	9.103 (0.043)	0.0004 (0.0000)

12 or GED	47,801 (15.6%)	16.592 (0.033)	0.0013 (0.0000)
Some college	73,575 (24.1%)	25.342 (0.032)	0.0029 (0.0000)
College graduate	68,463 (22.4%)	35.841 (0.037)	0.0051 (0.0000)
Advanced degree	68,205 (22.3%)	40.990 (0.038)	0.0066 (0.0000)
Not specified	31,649 (10.4%)	22.756 (0.051)	0.0019 (0.0000)
**Annual income ($)**			
<10k	36,197 (11.8%)	12.081 (0.033)	0.0007 (0.0000)
10k-25k	31,509 (10.3%)	20.664 (0.045)	0.0021 (0.0000)
25-35k	20,614 (6.7%)	26.279 (0.061)	0.0032 (0.0000)
35-50k	23,686 (7.8%)	30.675 (0.060)	0.0042 (0.0000)
50k-75k	32,772 (10.7%)	35.139 (0.053)	0.0052 (0.0000)
75-100k	25,513 (8.4%)	37.649 (0.061)	0.0060 (0.0001)
100-150k	31,799 (10.4%)	39.529 (0.055)	0.0059 (0.0000)
150-200k	14,816 (4.9%)	40.191 (0.081)	0.0058 (0.0001)
>200k	20,280 (6.6%)	42.062 (0.070)	0.0057 (0.0001)
Not specified	68,440 (22.4%)	23.343 (0.033)	0.0025 (0.0000)

Table S3: Open and click rates by demographic subgroups across 30 issues of MMM. Standard error is given in brackets.

6. Content preferences by demographic subgroup

For 30 MMM issues sent between March 2020 and October 2021, editorial staff and a licensed physician manually coded articles by topic, such as a medical condition, COVID-19, or women's health. Most articles (92%) have up to two labels. The full list of labels and article counts is: lifestyle (47 articles), medical conditions (38), personal story (29), COVID-19 (28), public health (21), diversity (16), aging (16), women's health (15), innovation (15), precision medicine (13). Note that COVID-19 was separated out as a category because of the sheer number of COVID-specific articles that were published in 2020 and 2021 (41 compared to 39 for all other articles tagged as 'Medical Conditions'). Other infectious diseases were kept under 'Medical Conditions'.

To assess content preferences, we look at the article click rate by topic label and normalize these at the demographic subgroup level so that we have a measure of relative engagement within that subgroup. The click rate by topic was compared across subgroups using proportions z-tests. All results reported below are statistically significant at a p-value of 0.001 or lower, unless otherwise noted.

Differences in content preferences are most pronounced when grouping participants by self-identified race and ethnicity and age (Figure S1; Table S4). Black and African American participants appear to have a strong preference for personal stories and articles about diversity, closely followed by COVID information, which together account for 41% of article clicks. These topic categories are clicked on more than 3 times as often as the least favored topics of lifestyle and public health (7-8% each). Diversity content is also relatively popular with Hispanic, Latino and Spanish participants (10.3% of clicks), participants self-identifying with more than one race (10.2%) and Asian participants (9.8% of clicks). White participants click on diversity around

8.3% of the time and in relative terms this is the topic with the least engagement for this group. Articles about innovation are favored by Asian participants, accounting for 11% of clicks and ranking third most popular, whereas this topic is less popular (8.5-9.5% of clicks) for other groups (p=0.02 for upper bound). White participants are more likely to engage with content about aging accounting for 11.8% of clicks compared to 8.7-10.5% for other groups (p=0.003 for upper bound).

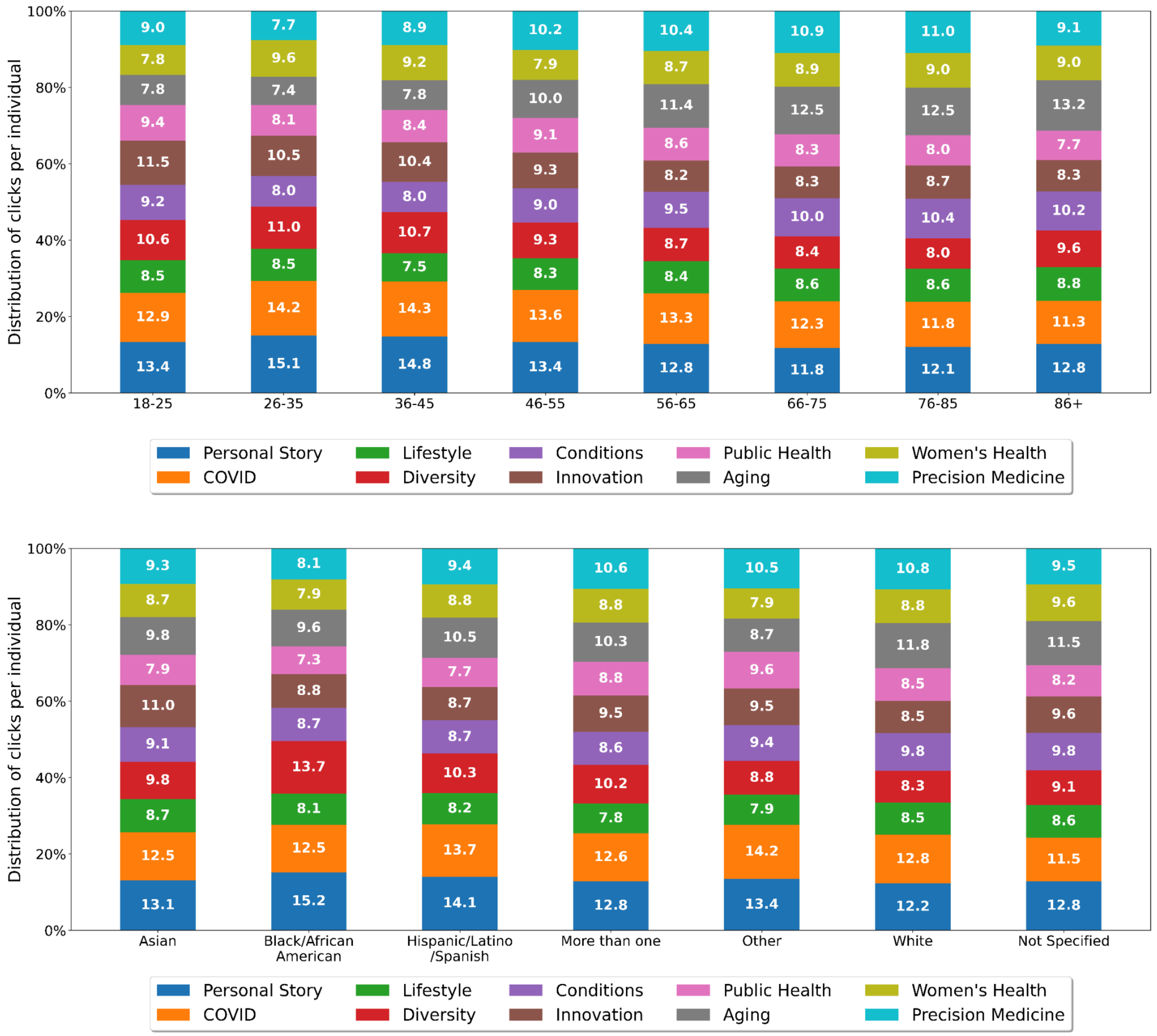

Figure S3: Distribution of clicks by topic, normalized at the subgroup level. Age is shown in the top panel and race/ethnicity in the bottom panel.

Looking at content preferences by age group, engagement with topics on aging and medical conditions increase with age and the differences are stark in relative terms (Figure S1). Aging is the least popular topic for participants aged 45 and younger[1] with 7.8% of clicks. The topic accounts for 10% of clicks for participants aged 46-55, increasing to 13% of clicks for participants aged 76-85, for whom it is the most popular topic[2]. Engagement with the articles about medical conditions also increases with age, accounting for 8% of clicks for those aged 45 and younger, 9% for those aged 46-55 (p=0.07) and 10.5% for those aged 76-85. Relative to other topics, articles about diversity and innovation are more of interest to participants aged 26-45 (accounting for 10-11% of clicks), compared to participants 56 and up (8-9% of clicks).

An important limitation of this analysis is that results are likely skewed by specific content placement (personal stories always feature as the first article) and timing of the analysis during the COVID-19 pandemic. For these reasons, our findings about the relatively high engagement rates for personal stories and COVID-19 content in particular should be interpreted with caution.

---

[1] Because of the low click rate and number of participants in the 18-25 group, we cannot definitively say that aging ranks at the bottom for this group because the confidence interval is very large. Directionally, however, the click rate is the lowest out of all the topic categories for this group along with women's health (7.8% of clicks).

[2] Directionally, aging is also the most popular topic for participants older than 86 years, but our confidence intervals are large such that we cannot definitively separate it from personal stories as for the most preferred topic.

	Click rate as a proportion of total clicks for that demographic subgroup (standard error in brackets)									
	**Personal story**	**COVID**	**Lifestyle**	**Diversity**	**Con-ditions**	**In-novation**	**Public health**	**Aging**	**Women's health**	**Precision medicine**
**Age**										
18-25	0.134 (0.009)	0.129 (0.009)	0.085 (0.006)	0.106 (0.012)	0.092 (0.007)	0.115 (0.012)	0.094 (0.011)	0.078 (0.01)	0.078 (0.01)	0.09 (0.013)
26-35	0.151 (0.004)	0.142 (0.004)	0.085 (0.002)	0.11 (0.005)	0.08 (0.003)	0.105 (0.005)	0.081 (0.004)	0.074 (0.004)	0.096 (0.005)	0.077 (0.004)
36-45	0.148 (0.003)	0.143 (0.003)	0.075 (0.002)	0.107 (0.004)	0.08 (0.002)	0.104 (0.004)	0.084 (0.003)	0.078 (0.003)	0.092 (0.004)	0.089 (0.004)
46-55	0.134 (0.002)	0.136 (0.003)	0.083 (0.002)	0.093 (0.003)	0.09 (0.002)	0.093 (0.003)	0.091 (0.002)	0.1 (0.003)	0.079 (0.003)	0.102 (0.003)
56-65	0.128 (0.002)	0.133 (0.002)	0.084 (0.001)	0.087 (0.002)	0.095 (0.001)	0.082 (0.002)	0.086 (0.001)	0.114 (0.002)	0.087 (0.002)	0.104 (0.002)
66-75	0.118 (0.001)	0.123 (0.001)	0.086 (0.001)	0.084 (0.001)	0.1 (0.001)	0.083 (0.001)	0.083 (0.001)	0.125 (0.002)	0.089 (0.001)	0.109 (0.002)
76-85	0.121 (0.002)	0.118 (0.002)	0.086 (0.001)	0.08 (0.002)	0.104 (0.001)	0.087 (0.002)	0.08 (0.002)	0.125 (0.002)	0.09 (0.002)	0.11 (0.002)
86+	0.128 (0.005)	0.113 (0.005)	0.088 (0.003)	0.096 (0.006)	0.102 (0.004)	0.083 (0.006)	0.077 (0.005)	0.132 (0.007)	0.09 (0.006)	0.091 (0.007)
**Gender identity**										
Man	0.116 (0.001)	0.128 (0.002)	0.086 (0.001)	0.094 (0.002)	0.098 (0.001)	0.096 (0.002)	0.092 (0.002)	0.115 (0.002)	0.072 (0.002)	0.103 (0.002)
Woman	0.129 (0.001)	0.128 (0.001)	0.084 (0.001)	0.086 (0.001)	0.096 (0.001)	0.083 (0.001)	0.082 (0.001)	0.114 (0.001)	0.093 (0.001)	0.106 (0.001)
Non-binary	0.141 (0.012)	0.137 (0.012)	0.079 (0.007)	0.098 (0.013)	0.077 (0.008)	0.108 (0.014)	0.086 (0.012)	0.099 (0.014)	0.094 (0.013)	0.081 (0.013)
Not specified	0.124 (0.004)	0.113 (0.004)	0.086 (0.003)	0.083 (0.004)	0.098 (0.003)	0.099 (0.005)	0.082 (0.004)	0.117 (0.005)	0.099 (0.005)	0.1 (0.005)
**Self-identified Race/Ethnicity**										
Asian	0.131 (0.005)	0.125 (0.005)	0.087 (0.003)	0.098 (0.006)	0.091 (0.003)	0.11 (0.006)	0.079 (0.005)	0.098 (0.005)	0.087 (0.005)	0.093 (0.006)
Black/ African American	0.152 (0.004)	0.125 (0.003)	0.081 (0.002)	0.137 (0.005)	0.087 (0.003)	0.088 (0.004)	0.073 (0.003)	0.096 (0.004)	0.079 (0.004)	0.081 (0.004)
Hispanic/ Latino/ Spanish	0.141 (0.004)	0.137 (0.004)	0.082 (0.002)	0.103 (0.004)	0.087 (0.002)	0.087 (0.004)	0.077 (0.003)	0.105 (0.004)	0.088 (0.004)	0.094 (0.004)
NHPI	0.124 (0.062)	0.194 (0.079)	0.077 (0.038)	0.114 (0.080)	0.071 (0.041)	0.178 (0.103)	0.128 (0.074)	0.114 (0.080)	0.000 (0.000)	0.000 (0.000)
More than one	0.128 (0.005)	0.126 (0.005)	0.078 (0.003)	0.102 (0.006)	0.086 (0.003)	0.095 (0.006)	0.088 (0.005)	0.103 (0.006)	0.088 (0.005)	0.106 (0.006)
Other race	0.134 (0.007)	0.142 (0.007)	0.079 (0.004)	0.088 (0.007)	0.094 (0.005)	0.095 (0.008)	0.096 (0.007)	0.087 (0.007)	0.080 (0.007)	0.106 (0.008)
White	0.122 (0.001)	0.128 (0.001)	0.085 (0.001)	0.083 (0.001)	0.098 (0.001)	0.085 (0.001)	0.085 (0.001)	0.118 (0.001)	0.088 (0.001)	0.108 (0.001)

Not specified	0.128 (0.004)	0.115 (0.003)	0.086 (0.002)	0.091 (0.004)	0.098 (0.003)	0.096 (0.005)	0.082 (0.003)	0.115 (0.005)	0.096 (0.004)	0.095 (0.005)
**Educational attainment**										
0-12	0.152 (0.011)	0.141 (0.011)	0.083 (0.007)	0.087 (0.011)	0.092 (0.008)	0.102 (0.012)	0.072 (0.009)	0.108 (0.013)	0.095 (0.013)	0.07 (0.011)
12 or GED	0.135 (0.003)	0.134 (0.003)	0.077 (0.002)	0.09 (0.004)	0.097 (0.002)	0.085 (0.004)	0.085 (0.003)	0.104 (0.004)	0.084 (0.004)	0.11 (0.004)
Some college	0.13 (0.002)	0.13 (0.002)	0.079 (0.001)	0.088 (0.002)	0.096 (0.001)	0.085 (0.002)	0.087 (0.002)	0.107 (0.002)	0.088 (0.002)	0.11 (0.002)
College graduate	0.124 (0.001)	0.129 (0.001)	0.085 (0.001)	0.087 (0.001)	0.097 (0.001)	0.085 (0.001)	0.083 (0.001)	0.116 (0.002)	0.089 (0.002)	0.104 (0.002)
Advanced degree	0.122 (0.001)	0.125 (0.001)	0.088 (0.001)	0.089 (0.001)	0.096 (0.001)	0.089 (0.001)	0.084 (0.001)	0.119 (0.002)	0.086 (0.001)	0.102 (0.002)
Not specified	0.125 (0.004)	0.113 (0.004)	0.085 (0.003)	0.084 (0.004)	0.096 (0.003)	0.1 (0.005)	0.084 (0.004)	0.115 (0.005)	0.098 (0.005)	0.102 (0.005)
**Annual income ($)**										
<10k	0.138 (0.005)	0.133 (0.005)	0.082 (0.003)	0.107 (0.006)	0.089 (0.004)	0.096 (0.006)	0.092 (0.005)	0.091 (0.005)	0.081 (0.005)	0.091 (0.006)
10k-25k	0.137 (0.003)	0.133 (0.003)	0.079 (0.002)	0.092 (0.003)	0.090 (0.002)	0.088 (0.003)	0.083 (0.003)	0.105 (0.004)	0.087 (0.003)	0.108 (0.004)
25-35k	0.131 (0.003)	0.127 (0.003)	0.083 (0.002)	0.094 (0.004)	0.093 (0.002)	0.085 (0.003)	0.086 (0.003)	0.108 (0.004)	0.084 (0.003)	0.109 (0.004)
35-50k	0.124 (0.002)	0.126 (0.002)	0.085 (0.002)	0.092 (0.003)	0.096 (0.002)	0.087 (0.003)	0.083 (0.002)	0.115 (0.003)	0.086 (0.003)	0.106 (0.003)
50k-75k	0.125 (0.002)	0.125 (0.002)	0.085 (0.001)	0.088 (0.002)	0.097 (0.001)	0.086 (0.002)	0.082 (0.002)	0.115 (0.002)	0.090 (0.002)	0.107 (0.003)
75-100k	0.125 (0.002)	0.127 (0.002)	0.085 (0.001)	0.087 (0.002)	0.098 (0.002)	0.085 (0.002)	0.084 (0.002)	0.118 (0.003)	0.089 (0.002)	0.104 (0.003)
100-150k	0.124 (0.002)	0.130 (0.002)	0.084 (0.001)	0.085 (0.002)	0.098 (0.001)	0.084 (0.002)	0.086 (0.002)	0.117 (0.002)	0.087 (0.002)	0.103 (0.002)
150-200k	0.124 (0.003)	0.129 (0.003)	0.086 (0.002)	0.086 (0.003)	0.097 (0.002)	0.086 (0.003)	0.084 (0.003)	0.117 (0.003)	0.087 (0.003)	0.102 (0.004)
>200k	0.120 (0.002)	0.132 (0.003)	0.084 (0.002)	0.087 (0.003)	0.096 (0.002)	0.090 (0.003)	0.088 (0.002)	0.113 (0.003)	0.085 (0.003)	0.106 (0.003)
Not specified	0.126 (0.002)	0.121 (0.002)	0.086 (0.001)	0.083 (0.002)	0.098 (0.002)	0.090 (0.002)	0.081 (0.002)	0.119 (0.003)	0.094 (0.002)	0.102 (0.003)

Table S4: Content preferences across demographic subgroups across 30 issues of MMM. The numbers represent the proportion of clicks by that subgroup on articles in a certain category. The standard error is given in brackets.

7. Intervention details and additional analysis results for the RCT featuring topics in the email subject line

Group	N *	Send 1	Send 2	Send 3
Control	90,429	“Week of November 2”	“Week of November 23”	“Week of February 8”
Intervention 1	90,434	“COVID, cognitive health and more”	COVID vaccines, lung cancer and more	“Precision medicine, mood boost and more”
Intervention 2	90,454	“Cognitive health, COVID and more”	“Lung cancer, COVID vaccines and more”	“Mood boost, precision medicine and more”
**Total**	**271,317**			

* Note that our N decreases by send because of participants unsubscribing from the newsletter. By the second send our sample size is 90,000 for control, treatment, order 1 = 90,028 for intervention group 1,and 90,031 for intervention group 2. For the third send, these counts are 87,250 for control, 87,278 for intervention group 1, and 87,207 for intervention group 2.

Table S5: Newsletter subject lines by experimental group

Topic in subject line *	Overall effect on open rate	Demographic with ex ante high engagement	N in demographic subgroup	Local effect for subgroup
Cognitive health (issue 1)	- 1.01 pp	Self-identified race/ethnicity: White	144,349	-1.35 pp
		Age: 56 years or older	127,581	-1.50 pp
		Education: College or advanced degree	121,307	-1.11 pp
		Income: $35,000 or higher	132,989	-1.27 pp
Lung cancer (issue 2)	- 0.8 pp	Self-identified race/ethnicity: White	143,816	-0.76 pp
		Age: 66 years or older	72,389	-1.46 pp
Precision medicine (issue 3)	-2.16 pp	Self-identified race/ethnicity: White	139,754	-2.69 pp
		Age: 46 years or older	165,924	-2.66 pp

Note that COVID was also included as one of the topics in the subject line for two issues. However, the predictive analysis finds that COVID was a topic of interest across all demographic subgroups, typically scoring as the most or second-most clicked content category. As such, we do not do additional subgroup analysis for this topic.

Table S6: Effect of including topics in the email subject lines for participants overall and for specific demographic subgroups.
